# Supplementary material for: The Role of CRP POC Testing in the Fight against Antibiotic Overuse in European Primary Care: Recommendations from a European Expert Panel
Source: Diagnostics (Basel). 2023 Jan 15;13(2):320. doi: 10.3390/diagnostics13020320 (PMC9857389; doi:10.3390/diagnostics13020320)
Supplement: Supplementary file 1 [file diagnostics-13-00320-s001.zip › diagnostics-2092837-supplementary.pdf]

This document contains the meeting schedule and the questions that have been asked to the expert panel.

CRP PoC Advisory Board - pre-meeting online survey

January 2022

New and established roles of CRP Point-of-Care testing in primary care (10-15 minutes)

Part I – CRP Point-of-Care testing (POCT) and Antibiotic prescribing

Part II – Role of CRP POCT for COVID-19

Part III – CRP POCT and indications beside RTIs

QUESTIONNAIRE:

Part I – CRP Point-of-Care testing (POCT) and Antibiotic prescribing

Q1. In your country, do you know the approximate % of antibiotic prescriptions for Respiratory Tract Infections (RTIs) in primary care and what % would be desirable from your point of view?

% prescriptions	
% desirable prescriptions	

Q2. According to your experience, what are the top 3 reasons for physicians to over prescribe antibiotics for RTI symptoms? Please, list the Top 3 reasons.

Q3. There are several strategies and tests to reduce antibiotics prescription in cases of RTIs in primary care. In what % of cases have you used the strategy in the past 12 months?

How likely would you recommend their usage? Please, provide a rating on a scale of 1 to 5 (where 1 being "Not recommend at all" and 5 being "highly likely to recommend").

Strategy in primary care		Strategy used in % of cases in the past 12 months	Rating (1-5)
1	Delayed prescribing		
2	Communication training of physicians		
3	Quantitative CRP Point-of-Care test		

4	Semi-quantitative CRP Point-of-Care test		
5	Simultaneous qualitative measurement of CRP (≥20 mg/L) and myxovirus resistance protein A (MxA) (≥40 ng/mL)		
6	Procalcitonin (PCT) test		
7	Influenza A&B test during the influenza season		
8	Strep A test for sore throat		
9	SARS-CoV-2 test		
10	Other (please, specify)		

Q4. Considering each strategy to reduce antibiotics prescription in cases of RTIs, how much do you agree with the following statements? Please, provide a rating on a scale of 1 to 5 (where 1 being "Strongly disagree" and 5 being "Strongly agree").

Statement		Communication training of physicians	Delayed prescribing	Quantitative CRP	Semi-quantitative CRP	Simultaneous qualitative CRP and MxA	PCT test	Influenza A&B during the influenza season	Strep A for sore throat	SARS-CoV-2	Other
1.	It is reliable/proven: robust scientific evidence, it helps reduce antibiotics prescription										
2.	It is innovative										
3.	It is safe										
4.	It is easy to use/perform										
5.	I am very familiar with it										
6.	It is cost-effective										
7.	It is easily accepted by the patient										

Q5. According to your perception and considering the strategies available, what are the top 3 barriers that prevent the reduction in antibiotics prescription in the case of RTIs?

Q6. Is CRP still suitable to rule out the need of antibiotic prescribing in RTIs during and after the ongoing COVID-19 pandemic?

**Statement**		**Strongly disagree**	**Disagree**	**Neither disagree or agree**	**Agree**	**Strongly agree**
1.	CRP has a role of ruling out the need of antibiotics for RTIs during and after the COVID-19 pandemic.					
2.	This is also true without knowing the COVID-19 infection status of a patient					

Q7. When should a CRP POC test be performed in patients presenting with symptoms of RTIs, when the physician is uncertain or always when he/she intends to prescribe antibiotics?

**Statement**		**Strongly disagree**	**Disagree**	**Neither disagree or agree**	**Agree**	**Strongly agree**
1.	A CRP POC test should be performed when the physician is uncertain to prescribe antibiotics for RTIs.					
2.	A CRP POC test should be performed once the physician intends to prescribe antibiotics for RTIs.					

Q8. According to your perception, how will antibiotics prescription be managed in 5 years from now in cases of RTIs?

a. **What changes do you foresee in the usage of CRP POC testing for reducing antibiotics prescription in cases of RTIs?**

Q9. What is your opinion about the monitoring with CRP POCT of patients taking antibiotics, to decide about the appropriate termination of antibiotic intake? Would this contribute to reduce the risk of antibiotic resistance?

**Statement**		**Strongly disagree**	**Disagree**	**Neither disagree or agree**	**Agree**	**Strongly agree**
1.	A CRP POC testing has a role in monitoring the effectiveness of antibiotics					
2.	A CRP POC test has a role in deciding about the termination of antibiotic intake					

Part II – Role of CRP POCT in COVID-19 testing

Q10. On a scale of 1 to 5, how much do you agree with CRP POC testing being adopted in GP's practices in patients presenting with SARS-CoV-2 to predict severity, mortality of the patient and assist in decision-making for hospital referral?

1	Strongly disagree
2	Disagree
3	Neither agree nor disagree
4	Agree
5	Strongly agree

Q11. On a scale of 1 to 5, how likely will CRP POC testing become adopted in GP's practices in patients presenting with SARS-CoV-2 to predict severity, mortality of the patient and assist in decision-making for hospital referral?

1	Highly unlikely
2	Unlikely
3	Somewhat likely
4	Likely
5	Highly likely

Part III – CRP POCT and indications beside RTIs

Q12. Are there any other indications in primary care setting beside RTIs where CRP POC testing would be suitable for? Please, list the top 3 indications for those you see a special advantage to have CRP POCT available at primary care.

Q13. What do you think about having CRP and hemoglobin available on the same POC platform in primary care? Please, list the top 3 indications for those you see a special advantage to have CRP and Hb POCT available at primary care.

CRP PoC 2nd Advisory Board - pre-meeting online survey

February 2022

New and established roles of CRP Point-of-Care testing in primary care
(10-15 minutes)

QUESTIONNAIRE:

Actions and initiatives to reduce antibiotics prescription and increase use of CRP POCT (15 mins)

Q1. Can you please outline the most common operational process for CRP testing in your country of practice? Feel free to refer to the below examples.

Example 1:

- During a consultation, a GP decides to perform a CRP test and takes a venous blood sample.
- The GP sends the sample to the lab for results.
- The next day, test results are sent back to the GP.
- The GP calls the patient to give a recommendation for treatment.

Example 2:

- During a consultation, a GP decides to perform a CRP POCT.
- The GP performs a CRP POCT and results are available within 10-15 minutes.
- The patient waits at the GP practice.
- The GP decides on treatment.

Example 3:

- CRP is usually not tested by the GP.
- The GP decides on treatment without CRP testing.

Q2. Can you please outline the financial process for CRP POCT in your country of practice? Feel free to refer to the below examples.

Example 1:

- A lab purchases a number of CRP POCT analyzers.
- The lab places a CRP POCT machine in a number of GP practices.
- The lab pays for disposables and manages supply and delivery.
- For each POCT performed, the lab gets reimbursed and pays a service fee to the GP.

Example 2:

- A GP purchases a CRP POCT analyzers to be placed in his/her practice.
- The GP pays for disposables and manages supply.
- For each CRP POCT performed, the GP gets reimbursed.

Example 3:

- A lab purchases a number of CRP POCT analyzers.
- The lab places a CRP POCT machine in a number of GP practices.
- The lab pays for disposables and manages supply and delivery.
- There is no reimbursement for each CRP POCT.

Example 4:

- A GP purchases a CRP POCT analyzers to be placed in his/her practice.
- The GP pays for disposables and manages supply.
- There is no reimbursement for each CRP POCT.

Q3. If we want to leverage CRP POCT to reduce antibiotics over prescription, what needs to happen? Please, list the TOP 3 items that you believe are necessary

Q4. Please rate how much you agree with each of the following statements.
In order to leverage CRP POCT to reduce antibiotics over prescription, we need to…

**Initiative/Action**		**Strongly disagree**	**Disagree**	**Neither disagree or agree**	**Agree**	**Strongly agree**
1.	Reinforce the place of CRP POCT in newly published European/International guidelines					
2.	Set in place a new incentivization (reimbursement/penalization) structure for the use of CRP POCT					
3.	Build further clinical evidence and data on the role of CRP POCT in low RTIs					
4.	Perform additional European research projects/studies to understand the barriers to reduce antibiotics prescription					

5.	Strengthen the collaboration with GP/Primary Care providers networks					
6.	Collaborate with NGOs or governmental organizations					
7.	Collaborate with industry partners or other sectors					
8.	Increase Direct to Patient Communication					
9.	Increase GP/PCP Education on antibiotics prescription					

Guidelines

a. Pre COVID-19 pandemic, what % of GP/PCPs would you estimate were following the guidelines for use of CRP POCT in LRTIs in your country?

b. Currently, what % of GP/PCPs would you estimate are following the guidelines for use of CRP POCT in LRTIs in your country?

c. EU Guidelines have recommended the use of CRP POCT since 2011.
What are the TOP 3 reasons adherence to guidelines is not higher?

d. Who are the stakeholders that need to be involved in the process of updating the guidelines? Please, list all the relevant stakeholders

Incentivization structure

a. How would an ideal incentivization structure (reimbursement/penalty) look to incentivize correct use of CRP POCT and subsequent antibiotics prescriptions for LRTIs?

Research/projects

a. What research topics or information do we need to better define an approach to reduce antibiotics over prescription for LRTIs?

GP/PCP Networks

a. Which GP/PCP networks would have to be leveraged to reduce antibiotics over prescription for LRTIs?

Other groups, sectors

a. Which NGOs or governmental organizations would have to be leveraged to reduce antibiotics over prescription for LRTIs?

b. Which other sectors or industry actors would have to be leveraged to reduce antibiotics over prescription for LRTIs?

Education/Communication

a. What topics would patients need to be aware of?

b. What topics would GP/PCPs need to be trained on?
